# Methylation of the Sox9 and Oct4 promoters and its correlation with gene expression during testicular development in the laboratory mouse

**DOI:** 10.1590/1678-4685-GMB-2015-0172

**Published:** 2016-07-04

**Authors:** Mamta Pamnani, Puja Sinha, Alka Singh, Seema Nara, Manisha Sachan

**Affiliations:** Department of Biotechnology, Motilal Nehru National Institute of Technology, Allahabad, 211004, India

**Keywords:** DNA methylation and development, Oct4, promoter hypermethylation, Sox9

## Abstract

Sox9 and Oct4 are two important regulatory factors involved in mammalian development. Sox9, a member of the group E Sox transcription factor family, has a crucial role in the development of the genitourinary system, while Oct4, commonly known as octamer binding transcription factor 4, belongs to class V of the transcription family. The expression of these two proteins exhibits a dynamic pattern with regard to their expression sites and levels. The aim of this study was to investigate the role of *de novo* methylation in the regulation of the tissue- and site-specific expression of these proteins. The dynamics of the *de novo* methylation of 15 CpGs and six CpGs in Sox9 and Oct4 respectively, was studied with sodium bisulfite genomic DNA sequencing in mouse testis at different developmental stages. Consistent methylation of three CpGs was observed in adult ovary in which the expression of Sox9 was feeble, while the level of methylation in somatic tissue was greater in Oct4 compared to germinal tissue. The promoter-chromatin status of Sox9 was also studied with a chromatin immune-precipitation assay.

## Introduction

Sox9 and Oct4 are two developmentally important genes that show differential spatial and temporal expression. SRY (sex determining region Y) box 9 (Sox9), located on chromosome 11 possesses a Sry-box which is the first gene to be expressed shortly after Sry gene expression (Chaboissier *et al.*, 2004; Koopman 2005; Vidal *et al.*, 2001). Sox9 has a crucial role in the development of the genitourinary system (Kent *et al.*, 1996). Sox9 expression was identified in the genital ridges of male and female sex gonads in 11.5 days post-coitum (dpc) embryos (Chaboissier *et al.*, 2004; Sekido *et al.*, 2004). Expression continues up to embryonic day 12.5, i.e. during testicular cord formation which later develops into seminiferous tubules, a signal for male gonad development. After day 12.5, expression of Sox9 is totally restricted to Sertoli cells and maintained until the adult stage (Kobayashi *et al.*, 2005; Koopman, 2005). RT-PCR and immunoblot identified Sox9 expression in mature adult testis but not in adult ovary (Kent *et al.*, 1996). Sox9 is an essential gene for testicular determination.

Oct4, commonly known as octamer binding transcription factor 4, belongs to class V of the transcription family present on chromosome 17. Oct4 is fully expressed in pre- implantation stage, oocytes, early embryonic pluripotent cells, adult germ cells and embryonic carcinoma cells, but later this expression is strictly limited to the inner cell mass (ICM) of the blastocyst and, following implantation, is confined to the epiblast (He *et al.*, 2009).

Germ cell-specific expression of Oct4 persists during the primordial germ cell migration in both sexes up to 13.5 dpc. The expression is down-regulated in female germ cells during zygotene and pachytene stage, but expression is still maintained in male germ cells. After birth, expression is up-regulated in primordial follicles at 12-14 days post-partum (dpp) and in mature oocytes (Ketkar and Reddy, 2012). Oct4 expression is absent in most somatic tissues, e.g. brain, muscle, liver, heart and intestine (Adachi *et al.*, 2007).

Since methylation can affect gene expression in various tissues (Sachan *et al.*, 2006; Pamnani *et al.*, 2014), in this study, we investigated the status of *de novo* methylation and expression pattern in the promoter region of mouse Sox9 and Oct4 gene during development. The correlation between the expression of these genes in the different developmental stages of testis and somatic tissues and methylation was also examined. Fifteen sites for Sox9 gene and six sites for Oct4 gene was investigated in somatic and germinal tissues of different developmental stages.

## Materials and Methods

### Genomic DNA extraction

Parkes strain of mice of both sexes was used in this study and the experimental protocols were approved by the institutional ethics committee. The mice were housed in groups of 6-7 per polypropylene cage (43 × 27 × 15 cm) under standard laboratory conditions. Mating was achieved by housing one male mouse with two female mice in separate cages. Pregnant females were isolated and housed separately after the detection of vaginal plug the next morning.

When required, the mice were killed by cervical dislocation and selected germinal and somatic tissues were excised, blotted free of blood and weighed. DNA was isolated from embryonic, neonatal and adult testes. Kidney and ovary were used as a source of somatic tissue DNA. Genomic DNA from adult and neonatal stages was isolated from three independent mice. Multiple MGCs (mesonephron gonadal complexes) were pooled from different embryos of the same stage, e.g. 11.5 dpc and 18.5 dpc to minimize the chance of contamination from other tissues. DNA was isolated with the help of standard protocol using Proteinase K digestion (50 μg/mL) at 37°C for 12-14 h. Phenol: chloroform: isoamylalcohol (25:24:1) extraction was done at 25°C. Finally, DNA was precipitated with 1/30 volume of 3 M sodium acetate (pH 5.0) and two volumes of chilled absolute ethanol.

### Sodium bisulfite treatment

The Bisulfite conversion was carried out according to the protocol of Clark *et al.* (1994), with minor modifications. Briefly, 1-2 μg DNA in a volume of 50 μL was denatured by adding 3 μL of 5 M NaOH and incubating for 15 minutes at 37°C. After denaturation, 420 μL of 3.9 M (saturated) sodium bisulfite (Sigma, final concentration 3.4 M; pH 5.0) and 33 μL of 20 mM hydroquinone (Sigma, final concentration 0.58 mM) were added to the denatured DNA and incubated at 50°C for 12-14 h. Treated DNA was desalted and purified using the Wizard DNA Clean-Up system (Promega, USA), desulfonated by adding 3 μL of 5 M freshly prepared NaOH, followed by incubation for 15 minutes at 37°C and finally precipitated with 1 μL of glycogen (Fermentas, final concentration 150 μg/mL, 25 μL of 10 M ammonium acetate (pH 7.0) and 150 μL of chilled absolute ethanol. The precipitated DNA was pelleted and resuspended in 40 μL of sterile water and stored at -20 °C until used.

### PCR of bisulfite-treated DNA

**Figure 1:**
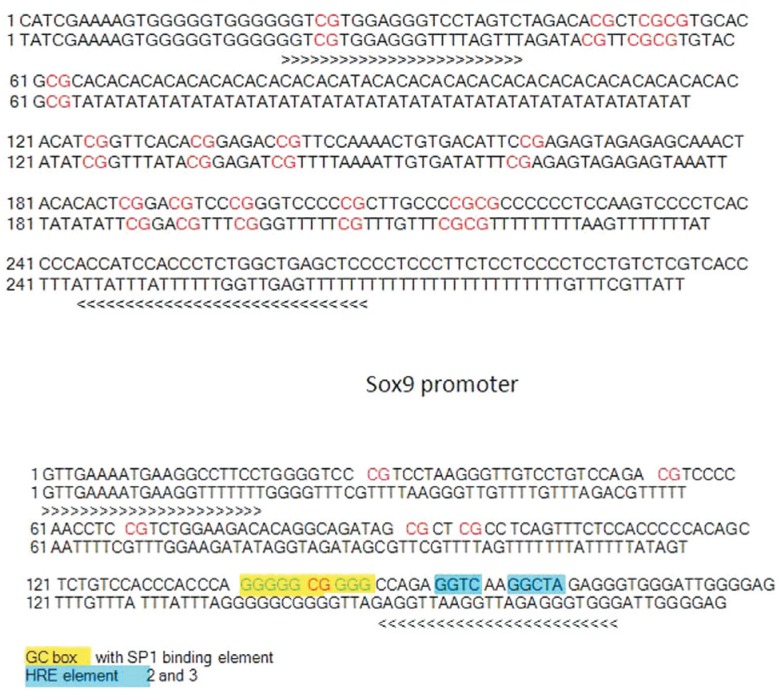
The complete bisulfite converted nucleotide sequence of the 5'UTR of Sox9 and Oct4 genes. The 250 bp region in the 5’UTR of Sox9 (harboring 15 CpG sites) and 145 bp regions in the promoter of Oct4 (harboring 06 CpG sites) were analyzed for their methylation pattern. CpG sites are highlighted in red. The locations of transcription factor binding sites are shown in different colors (yellow and blue) in Oct4 sequence.

Approximately 100-150 ng of bisulfite-treated DNA was used for each PCR. HotStar *Taq* polymerse (Qiagen) was used for amplification of bisulfite-converted DNA. PCR conditions were 94°C for 4 min, followed by 94°C for 1 min, 60°C for 1 min for Sox9 and 58°C for Oct4 and 72°C for 1 min for 35 cycles with a final extension at 72°C for 6 min. The primer pairs were used to amplify the 250 bp region (from base pairs 1 to 250) for Sox9 and the 145 bp region (from base pairs 2641 to 2786) for Oct4 gene. The primers sequences of Sox9 are FP: 5-GTTGTGGAGGG TTTTAGTTTAGATA-3 and RP 5'-AAAAAAAACTC AACCAAAAAA TAAATAATA-3; Oct4 gene FP: 5'-GTTGAAAATGAAGGTTTTTTTGG-3', RP 5'-CCACC CTCTAACCTTAACTCCTAAC-3'. The GenBank accession numbers for Sox9 and Oct4 gene are AB022193.1 and AJ297528.1 respectively. The promoter sequences and the location of CpGs are shown in Figure 1.

### Quantitative RT-PCR

Total cellular RNA was extracted using QIAzol reagent (Qiagen) followed by chloroform extraction. RNA was precipitated with isopropanol and dissolved in DEPC (diethyl pyro-carbonate) water. The concentration of RNA was determined spectrophotometrically (OD_260_nm) both before and after DNAse treatment. Equal amount of the total RNA was reverse transcribed using oligo-dT primers and 200U of MMLV Reverse transcriptase (Fermentas). PCR amplification was done with Maxima SYBR Green/ROX qPCR Master Mix (Thermo Scientific USA) in real time PCR machine (ABI-7500). Reactions were performed in triplicates for the housekeeping gene (GAPDH) and target gene (Sox9 and Oct4) for each developmental stage. The average of the cycle threshold (Ct) values and standard deviation were determined. Target mRNA amount was determined and normalized relative to the amount of GAPDH mRNA. ΔCt value was calculated by subtracting Ct value of reference gene (GAPDH) from Ct value of target gene (Sox9 and Oct4). For calculating ΔΔCt, ΔCt value of calibrator was subtracted from ΔCt value of each sample. Fold change in gene expression was calculated according to 2^-ΔΔCt^ method(Livak and Schmittgen, 2001).

The sequences of the primers are as follows: Sox9 FP: 5-GTGGCAAGTATTGGTCAA-3 and RP: 5-GAACAGA CTCACATCTCT-3; Oct4 FP: 5-GGCGTTCGCTTTGG AAAGGT GTC-3 and RP: 5-CTCGAACCACATCCTCT CT-3 and GAPDH FP: 5'-GGAGCCAAACGGGTCAT CATCTC-3' and RP: 5'-GAGGGGCCATCCACAGTCT TCT-3'. PCR conditions were as follows: 94°C for 4 min, followed by 40 cycles of denaturing at 94°C for 1 min, annealing at 60°C for 1 min, extension at 72°C for 1 min and final extension at 72°C for 6 min. Semi-quantitative RT-PCR was performed with same primer pair and following same PCR conditions except the cycle number (36 cycles for Sox9 and 35 cycles for Oct4 and GAPDH).

### Cloning and sequencing

Amplified PCR products were purified using Gel extraction kit (Qiagen). The purified products were cloned in T-vector using an InsTAclone™ PCR cloning kit (Thermo Scientific USA). Positive clones were selected and plasmid isolation was carried out by alkaline lysis method (Plasmid Miniprep Kit, Thermo Scientific). Sequencing of 7-10 independent clones for each developmental stage were done by automated DNA sequencer (ABI 3130 genetic analyzer). 50-100 ng of plasmid DNA was used for sequencing with Big Dye^®^ Direct Cycle Sequencing Kit as per manufacturer instructions.

### Chromatin immunoprecipitation assay

Chromatin was isolated from adult testis and adult ovary. The excised tissues were homogenized and subjected to collagenase treatment (Life Technologies, 50-200U/mL), followed by incubation at 37°C for 2-6 h. The cells were then dispersed by passing through a sterile stainless steel or nylon mesh and cell counting was performed using haemocytometer. The minimum number of cells required to perform ChIP experiments is 1X10^6^cells. Cell cross-linking was done by adding 37% formaldehyde (final concentration 1%, w/v) and kept for 10 min at 25°C on a rotating wheel followed by quenching with 1.25M glycine (final concentration 125mM) for 5 min at 25°C. Cells were then centrifuged at 4ºC for 5-8 min. Supernatant was discarded and the cell pellet was resuspended in lysis buffer (containing protease inhibitors: leupeptin-10 μg/mL, aprotinin-10 μg/mL and PMSF-1 mM). The cell suspension was subjected to sonication using a sonicator (SKN-IIDN) at the rate of 3sec ON/1sec OFF for 3-4 cycles for obtaining the desired chromatin range from 200-800bp. The sheared chromatin was further processed for pre-clearing by adding an IP-incubation mix and pre-blocked beads. Antibodies specific for capturing the desired protein and the interacting DNA were used (H3K4me3, Diagenode MAb-152-050 and H3K9me3, Diagenode, MAb-146-050, concentration 1μg/μl). Negative control IgG antibody (Diagenode, C15400001 (C15200001) was used for immuno-precipitating non-specific target and the associated DNA fragments. Bead washing with wash buffer-1, 2 and 3 removes non-associated DNA fragments. Protein/DNA complexes were eluted from pre-blocked beads by the addition of elution buffer.

The eluted complex was reversibly cross-linked and purified using phenol: chloroform: iso-amyl alcohol. The DNA fragments were precipitated by adding DNA precipitant, DNA co-precipitant and chilled absolute ethanol. The DNA pellet was resuspended in 30μl of milliQ water and the relative amount of specifically immunoprecipitated DNA was analyzed through PCR amplification using quantitative real-time PCR (ABI 7500) with 1.0μl of DNA, Maxima SYBR green/ROX qPCR master mix 2X (Thermo Scientific) and gene specific primers. Control primers GAPDH (c17021045, Diagenode) were used as positive control against activated chromatin regions) and TSH2β (c17021042, Diagenode) were used as positive control against repressed chromatin regions. The percentage input and fold enrichment was calculated which represents the enrichment of certain histone modifications on specific chromatin region. The chromatin immunoprecipitation (ChIP) assay result was analyzed according to the manufacturers instructions (Diagenode ChIP kit, Cat. no. kch-orgHIS-012).

## Results

Sodium bisulfite genomic DNA sequencing and Real-time PCR were used to evaluate the pattern of *de novo* methylation and mRNA quantification of Sox9 and Oct4 gene in somatic and germinal tissues respectively during different developmental stages. GAPDH was used as internal control in Real-time PCR. Methylation pattern was analysed in the 248 bp promoter region of Sox9 gene, which contains 15 CpG sites and is located immediately upstream of transcription start site. A 153 bp core promoter region of Oct4, harboring six CpG sites was analysed. A minimum of seven clones were sequenced to check the level of methylation at each developmental stage. Figure 1 shows nucleotide sequence of Sox9 and Oct4 gene promoter.

### Methylation pattern

**Figure 2:**
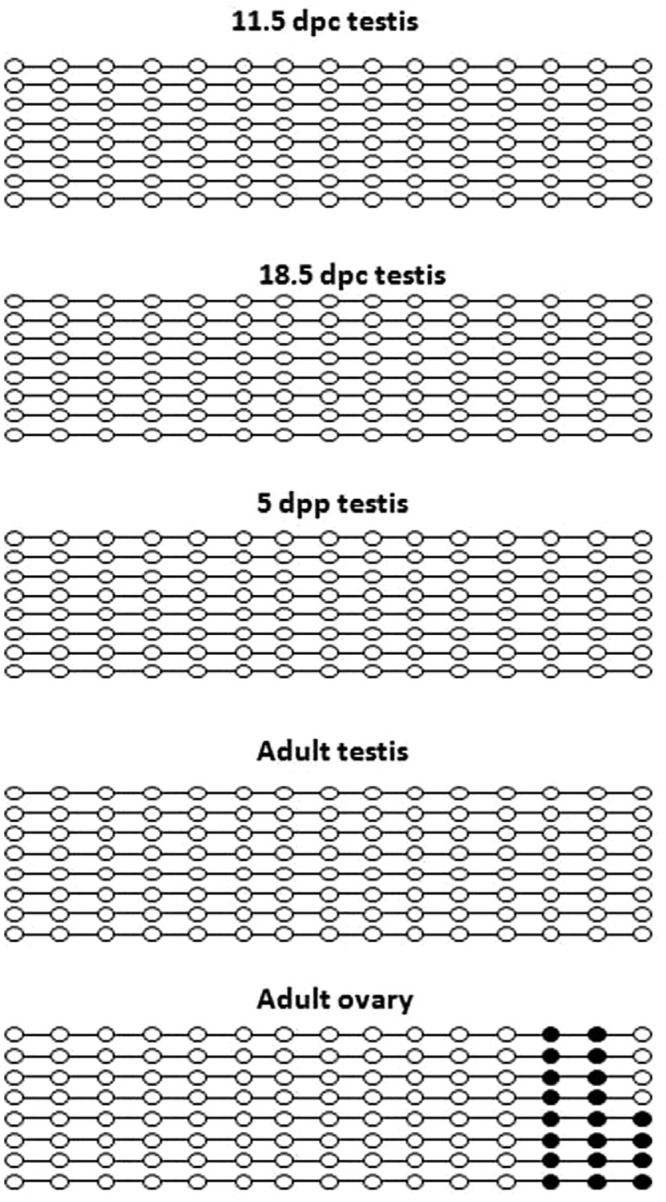
Methylation mapping of 15 CpGs (1-250 bp) in 5'UTR region of Sox9 gene in testicular tissue at different stages of mouse development (11.5 dpc, 18.5 dpc, 5 dpp, adult testis and adult ovary). Each line represents individual clone harboring 15 CpG sites. Methylated and unmethylated CpGs are represented by dark circles and white circles respectively.

**Figure 3:**
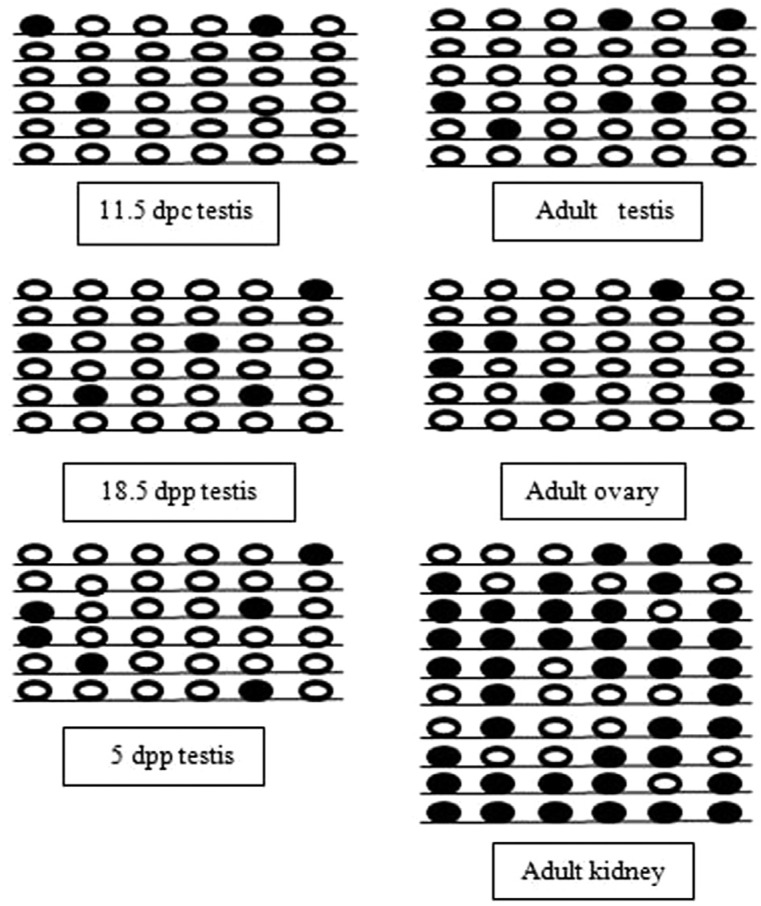
Methylation mapping of 6 CpGs (1-145 bp) in 5'UTR region of Oct4 gene in testicular tissues at different stages of mouse development (11.5 dpc, 18.5 dpc, 5 dpp, adult testis, adult ovary and adult kidney). Each line represents individual clone harboring 6 CpG sites. Methylated and unmethylated CpGs are represented by dark circles and white circles respectively.

Cytosines which show > 50% methylation were considered as methylated CpGs. In Sox9 gene, none of the site was methylated in fetal, neonatal and adult stages of testicular development (Figure 2). In adult ovary, CpG site 14 and 15 were fully methylated while site 16 showed 50% methylation (Figure 2). In case of Oct4 gene, average percentage methylation in testis at 11.5 dpc and 18.5 dpc was 19% whereas it was found to be 22% in the same tissue at 5 dpp. Average methylation in adult testis was 27% whereas adult ovary was methylated up to 31% (Figure 3). The highest percentage of average methylation (50%) was found in adult kidney (Figure 3). The Sp1 binding motif, harbors 6^th^ CpG site, was heavily methylated (67%) in adult kidney.

### Gene expression pattern

**Figure 4:**
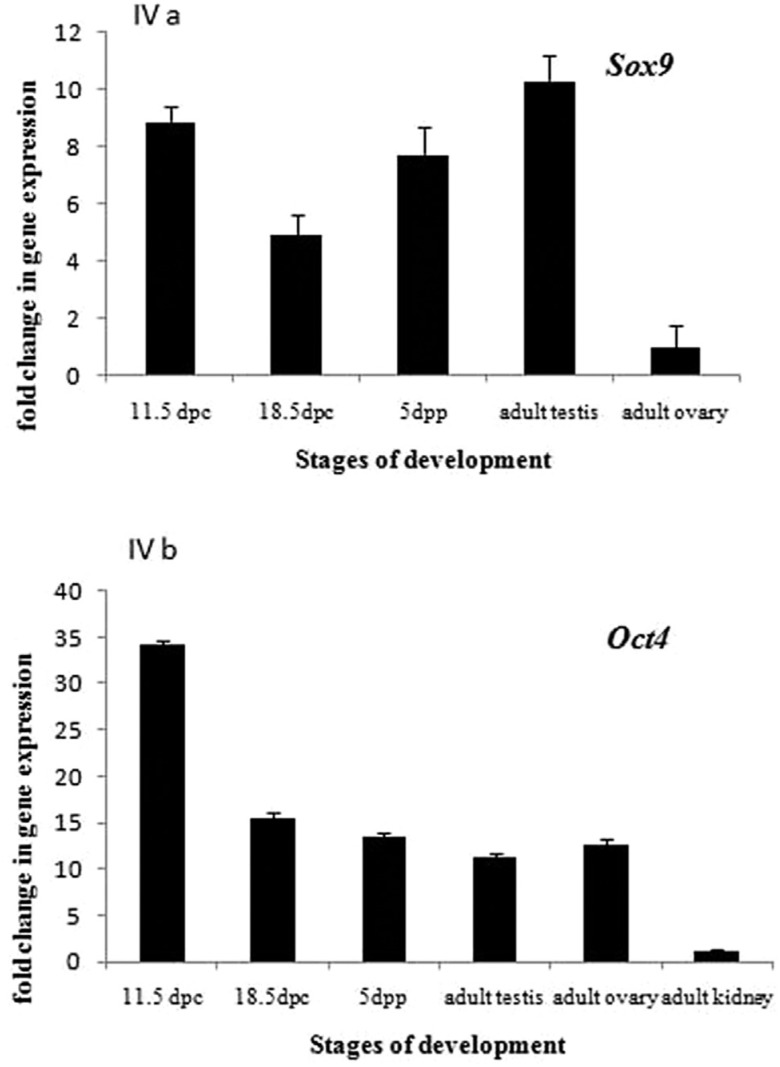
Expression pattern of Sox9 (**A**) and Oct4 (B) transcript in the testis at different developmental stages and in adult ovary and adult kidney (in Oct 4 only) through Real-time PCR (ΔCt for Sox9 and Oct4 was calculated for each stage in all tissues). GAPDH was taken as internal control. All results are shown as the means ± S.D and are considered significant at p ≤ 0.05. For calculating ΔΔCt, ΔCt value of calibrator was subtracted from ΔCt value of each sample. Fold change in gene expression was calculated according to 2^-ΔΔCt^ method.

Sox9 gene was expressed throughout testicular development, with little difference among the various stages (embryonic, fetal and neonatal). Embryonic and adult stages showed higher expression of Sox9, followed by neonatal and fetal stage (Figure 4A). Since Sox9 is one of the key players in testicular development, its expression in adult ovary was observed to be very low. Expression of Oct4 gene was highest in embryonic stage of testicular development (11.5 dpc). Fetal (18.5dpc), neonatal, adult testis and adult ovary showed comparable level of Oct4 expression. Virtually, no expression of Oct4 was observed in somatic tissue (adult kidney) (Figure 4B).

### Chromatin assembly data

**Figure 5:**
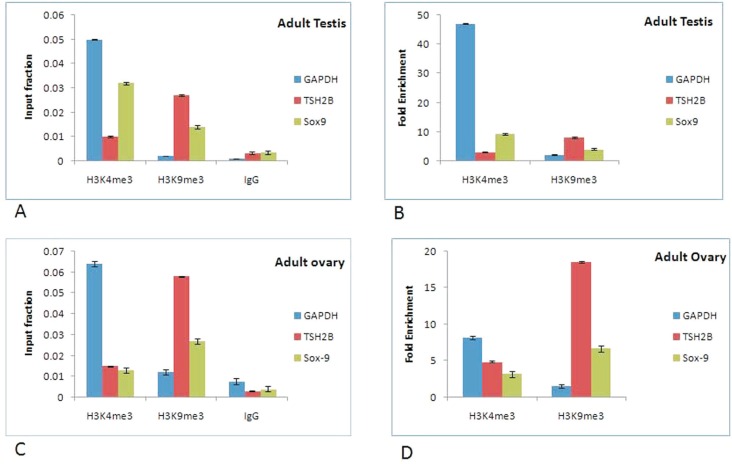
Percentage input and fold enrichment done by ChIP-qPCR to assess the H3K4me3 and H3K9me3 occupancy of Sox9 gene in (V a) & (V b) adult testis & (V c) & (V d) adult ovary. Error bars represents the means ± SD. Each data point represents the average of three independently prepared DNA samples immunoprecipitated with anti-H3K4me3 and anti-H3K9me3 antibodies.

The ChIP data for Sox9 shows that the input fraction and fold enrichment of Sox9 gene in adult testis was higher in activated regions of chromatin (immunoprecipitated with H3K4me3) as compared to repressed chromatin regions (immunoprecipitated with H3K9me3), while reverse was true in case of adult ovary in which the input fraction and fold enrichment was higher in repressed regions of chromatin (Figure 5A, C). The fold enrichment was approximately three times higher for activated regions of chromatin in adult testis as compared to adult ovary. However, fold enrichment of repressed chromatin state in adult ovary was 1.65 fold higher than in adult testis (Figure 5B, D). These data indicate that the chromatin around Sox9 gene remains active and continues to be expressed throughout testicular development. In contrast, ChIP data for ovary suggests that the chromatin around Sox9 gene remains partially inactive, thereby resulting in its reduced expression. In addition, the input fraction and fold enrichment was slightly higher in repressed region of chromatin in adult kidney as compared to adult testis for Oct4 (data not shown).

## Discussion

In present study, DNA methylation and expression profile of two developmentally important genes (Sox9 and Oct4) was studied during testicular development. Sox9 and Oct4 gene expression was examined in adult ovary and in different developmental stages (fetal, neonatal and adult) of testis, with Oct4 expression also being assessed in adult kidney.

During mouse gonadogenesis, Sox9 is detected in the male gonad at 11.5 dpc and in the testicular cords at 12.5 dpc, when male and female gonads can be morphologically distinguished. From this stage onwards, Sox9 expression is restricted to the Sertoli cells and persists in adult mice, suggesting its role in the regulation of germ cell differentiation (Fröjdman *et al.*, 2000; Kobayashi *et al.*, 2005). The expression profile of Sox9 in male and female gonads suggests that repression of Sox9 is critical for ovarian development (Swain *et al.*, 1998). Our quantitative Real-time PCR results indicate that although Sox9 was expressed throughout testicular development, its expression was highest in adult testis. Embryonic, fetal and neonatal stage showed almost equal expression, indicating its vital role during testicular development. In contrast, ovary showed lower expression compared to adult testis. Methylation was completely absent in fetal, neonatal and adult stages of testicular development, suggesting that the Sox-9 promoter remains active for the binding of transcription factors throughout development and tissue differentiation. Adult ovary showed 100% methylation at two sites and 50% methylation at one site. Even though methylation was completely absent at different stages of testicular development, the level of expression varied spatially and temporally. These results were confirmed by ChIP data analysis which showed that the input fraction and fold enrichment of activated chromatin was approximately two-fold higher than that of repressed chromatin, although the reverse was true in case of adult ovary, where the expression was highly compromised. This combination which involves activation and repression of chromatin modifications indicate that the methylation pattern established during development is profoundly important in determining the structural profile of gene expression.

Oct4 activates genes essential for murine embryonic stem cell survival and proliferation while selectively represses genes required for cell differentiation (Loh *et al.*, 2006). The epigenetic control of Oct4 expression in a stage- and cell type-specific manner during early embryogenesis is regulated by the hyper/hypomethylated status of the enhancer/promoter region (Hattori *et al.*, 2004). We observed an inconsistent heterogeneous methylation pattern throughout the development of testicular tissue. Marikawa *et al.* (2005) examined the DNA methylation pattern of the Oct4 regulatory element in P19 embryonic carcinoma cells, NIH3T3 embryonic fibroblasts and in adult somatic tissues such as liver, spleen and cumulus cells. The regulatory element was unmethylated in P19 embryonic carcinoma cells which strongly express Oct4 but markedly methylated in somatic cells. However, extent of methylation was heterogeneous in adult somatic cells. Luciferase reporter assay demonstrated that the extent of methylation directly affects the level of gene expression driven by the Oct4 regulatory element in P19 cells. Our results also indicate that the epigenetic status of Oct4 is heterogeneous among somatic cells, with average percentage methylation being higher in renal tissue than in testis and ovary. A progressive decline in Oct-4 expression was observed during testicular development from embryo to adult, while adult testis and ovary showed almost similar level of expression. Somatic tissues reflected higher level of methylation as compared to germinal tissues. Methylation of CpG sites adjacent to Sp1/Sp3 binding motif might affect the binding of Sp1 and hence could decrease the expression in adult kidney, a non-expressing tissue. It might be the common factor behind the absence of Oct4 expression in somatic tissues. Hypermethylation outside of the consensus Sp1/Sp3 element may interfere with Sp1/Sp3 binding as shown by an electrophoretic mobility shift assay although methylation within the consensus Sp1-binding site did not reduce Sp1/Sp3 binding (Zhu *et al.*, 2003). Binding of Sp3, another member of the Sp1 transcription factor family, to Oct4 promoter in embryonic stem cells suggests its complementary role with Sp1 in undifferentiated embryonic cells (Pesce *et al.*, 1999).

In conclusion, methylation of promoter/regulatory region is a crucial factor which directly affects Sox9 and Oct4 gene expression. As adult testis strongly expresses Sox9, site-specific methylation in adult ovary might be important in reducing Sox9 gene expression.
